# Unbalanced serum immunoglobulins in clinical subtypes of pediatric tuberculosis disease

**DOI:** 10.3389/fped.2022.908963

**Published:** 2022-08-09

**Authors:** Filippo Consonni, Nicolò Chiti, Silvia Ricci, Elisabetta Venturini, Clementina Canessa, Leila Bianchi, Francesca Lippi, Carlotta Montagnani, Mattia Giovannini, Elena Chiappini, Luisa Galli, Chiara Azzari, Lorenzo Lodi

**Affiliations:** ^1^Meyer Children's Hospital, Florence, Italy; ^2^Department of Health Sciences, University of Florence, Florence, Italy; ^3^Immunology Unit, Department of Pediatrics, Meyer Children's Hospital, Florence, Italy; ^4^Infectious Diseases Unit, Department of Pediatrics, Meyer Children's Hospital, Florence, Italy; ^5^Allergology Unit, Department of Pediatrics, Meyer Children's Hospital, Florence, Italy

**Keywords:** *Mycobacterium tuberculosis*, tuberculosis, children, immunoglobulins, humoral immunity, selective IgM deficiency, selective IgA deficiency

## Abstract

Immune response to tuberculosis (TB) has been extensively studied in the past decades and classically involves cellular immunity. However, evidence suggests that humoral immunity may play a relevant role. Past studies regarding serum immunoglobulin (Ig) levels in TB are dated and only involve adult subjects. In this study, we retrospectively studied a cohort of 256 children with TB disease and analyzed 111 patients screened for total serum Ig at diagnosis. According to the severity and extent of organ involvement, subjects were divided into four groups, namely, uncomplicated pulmonary TB (UCPTB, 56.3% of patients), complicated pulmonary TB (CPTB, 22.5%), lymph node extrapulmonary TB (LN-EPTB, 7.2%), and extra-nodal extrapulmonary TB (EN-EPTB, 13.5%). Serum IgG and IgA levels were significantly higher in more severe and extended TB disease. Median IgG levels progressively increased from uncomplicated to complicated pulmonary and nodal forms, reaching their highest values in diffuse extra-pulmonary TB. In parallel, UCPTB showed significantly lower frequencies of patients presenting a substantial increase in IgG levels when compared with the other three groups. No relevant differences in IgM levels were detected. Ig screening at follow-up showed a significant reduction in IgG and IgA levels. Finally, we unveiled three cases of selective IgA and one case of selective IgM deficiencies (SIgMD), the latter with a severe clinical course. Serum IgG and IgA may be a useful clinical tool to assess the severity and monitor the treatment response in pediatric TB disease. Moreover, immunological workup in children with TB disease may unmask primary defects of humoral immunity.

## Introduction

Tuberculosis (TB) is a major public health burden, accounting for 1.5 million deaths per year worldwide among both human immunodeficiency virus (HIV)-positive and HIV-negative patients. Notably, about 220,000 deaths occur in children (1). Immune response to *Mycobacterium tuberculosis* (MTB) has been extensively studied in the past decades (2, 3) and classically involves both macrophages and cellular immunity, which are paramount to generate tuberculous granulomas (4). However, B lymphocytes also participate in host defense against MTB, and humoral immunity is becoming an emerging player in the immune response against TB (5–7).

Primary defects of specific T lymphocyte pathways severely jeopardize host defense against *Mycobacteria* resulting in Mendelian susceptibilities to mycobacterial disease (MSMD) (8, 9). Moreover, secondary modifications of peripheral T cells are displayed both by adults and children with TB (10, 11). Symmetrically, humoral immunity also behaves in a similar fashion. Primary antibody deficiencies (e.g., Bruton's agammaglobulinemia and selective immunoglobulin M deficiency, SIgMD) have been associated with severe forms of mycobacteriosis (12–14). Moreover, secondary defects of specific B cell subsets have been reported in TB disease (15), especially in cases with severe lung involvement (16).

Despite the emerging role of humoral immunity, clinical studies regarding serum immunoglobulin (Ig) levels in TB patients are dated and only involve adult patients (17–25). Alterations of specific Ig classes may potentially correlate with the extent of the disease (19, 22). However, limited information about the humoral response in children is available (26, 27).

In this study, we evaluated serum IgG, IgA, and IgM levels in children affected by TB disease with different severity and organ involvement. In doing so, we investigated potential correlations between the extent of TB and secondary humoral alterations. Moreover, we aimed to unmask children with primary antibody deficiencies displaying TB as a first clinical manifestation of the underlying immune disorder.

## Methods

### Study population

We retrospectively identified all patients aged under 18 years, diagnosed with TB disease at Meyer Children's University Hospital (Florence, Italy) between 1 January 2004 and 1 May 2021 and screened for serum Ig levels at diagnosis (Ig screening was performed ±14 days from hospitalization for TB disease). Patients with known underlying causes of primary (inborn errors of immunity, IEI) or secondary immunodeficiency (e.g., HIV infection and immunosuppressive treatments) were excluded from the study. Selective deficiencies of one single Ig class (e.g., selective IgA deficiency, SIGAD; IgM deficiency, SIgMD) (28) in the absence of other major immunological defects were included in the study, and their clinical behavior was specifically described. We reviewed the clinical records and gathered demographic, clinical, and laboratory data including the localization and severity of TB, and serum Ig levels at diagnosis and, if available, at follow-up (i.e., 1–6 months after diagnosis).

### Disease group inclusion criteria

Tuberculosis disease was defined as the presence of at least one clinical specimen (gastric aspirate/lavage, sputum, or other samples) resulting positive for MTB on culture, microscopy, or nucleic acid amplification. Moreover, TB disease was also diagnosed in case of consistent clinical and radiological findings with either exposure to a known TB case or with a positive tuberculin skin test (TST) and/or interferon-gamma release assay (IGRA) (29). Treatment was started at diagnosis.

Children were classified into 4 groups based on the localization and severity of TB disease, namely, uncomplicated pulmonary TB (UCPTB), complicated pulmonary TB (CPTB), lymph node extrapulmonary TB (LN-EPTB), and extra-nodal extrapulmonary TB (EN-EPTB). UCPTB was considered in case of an isolated lung involvement in the absence of extensive cavitations, endobronchial localization, or massive pleural effusion. On the contrary, the presence of at least one of the three pulmonary features defined CPTB (11). A diagnosis of LN-EPTB was assigned to patients displaying extrapulmonary tuberculous lymphadenitis in the absence of other organ involvement. EN-EPTB was defined in case of miliary spread, disseminated TB, or involvement of extrapulmonary organs (e.g., central nervous system, bones, skin, and joints).

### Serum immunoglobulin analysis

Serum Ig levels were determined using a nephelometric assay (Dimension Vista^®^, Siemens, Munich, Germany) until 2019 and an immunoturbidimetric assay (Tina-quant^®^, Roche Diagnostics GmbH, Mannheim, Germany) from 2020 onward. Before the introduction of the new assay, the two methods were calibrated on multiple samples in order to align the results and maintain the same reference ranges. A group of healthy controls was not considered in the study design due to the availability of age and sex-specific reference ranges, to which patients' Ig levels were compared (30). Serum Ig levels at diagnosis were considered “elevated” or “reduced” if higher than two standard deviations (SD) or lower than −2 SD, respectively. Levels between 1 SD and 2 SD and between −1 SD and −2 SD were considered “borderline high” or “borderline low,” respectively. Serum Ig levels at follow-up were considered “increased” or “decreased” from diagnosis in case of a reduction >5% or an increase >5% of initial Ig levels, respectively. Ig levels at follow-up between −5 and 5% from diagnosis were considered “stable”.

### Statistical analysis

Statistical analyses were performed using GraphPad Prism (Version 9.0 for Mac, GraphPad Software, San Diego, CA, USA). Metric data were tested for normal distribution. Continuous variables were expressed as median values and interquartile ranges (IQRs). Student's *t*-test and Mann-Whitney test were used for the comparison of two groups displaying normal and non-normal distributions, respectively. Kruskal-Wallis test was used for the comparison of more than two groups. Categorical variables were expressed as numbers (%) and analyzed using chi-square test or Fisher's exact test, when appropriate. A *p*-value <0.05 was considered statistically significant.

## Results

### Composition of the study groups

A cohort of 256 children with TB disease was retrospectively screened for serum Ig levels at diagnosis (Supplementary Figure 1). A total of 111 children without underlying known causes of immunodeficiency and with at least one Ig detection available at diagnosis were included in the study (median age: 5.1 years, IQR 2.4–12.8; 41.4% females) and divided into four groups according to the localization and severity of TB disease. UCPTB was recorded in 63/111 patients (56.3%), CPTB in 25/111 patients (22.5%), LN-EPTB in 8/111 patients (7.2%), and EN-EPTB in 15/111 patients (13.5%). No statistically significant difference in terms of age and sex distribution was observed among the four groups. Demographic and clinical data are fully shown in Table 1.

**Table 1:** Demographic data and serum Ig levels at diagnosis in the four TB study groups.

**Ig at diagnosis**	**UCPTB**	**CPTB**	**LN-EPTB**	**EN-EPTB**	**Multiple comparison (Kruskal-Wallis) *p*-value**
	**(*n =* 63)**	**(*n =* 25)**	**(*n =* 8)**	**(*n =* 15)**	
**Female** *n* (%)	30 (47.6%)	8 (32.0%)	4 (50.0%)	4 (26.7%)	ns
**Age years** median (IQR**)**	4.0 (2.0–9.2)	8.5 (3.2–15.1)	9.9 (2.1–15.1)	8.6 (3.6–16.6)	ns
**IgG mg/dL** median (IQR)	1,070 (912–1,330)	1,360 (1,125–1,765)	1,510 (875–1,920)	1,748 (1,020–2,030)	0.0035
**IgA mg/dL** median (IQR)	110 (72–205)	230 (171–328)	153 (60–483)	196 (116–367)	0.0019
**IgM mg/dL** median (IQR)	121 (94–160)	131 (104–176)	128 (112–163)	116 (74–140)	ns

Ig, immunoglobulins; n, number of patients; IQR, interquartile range; M, male; F, female; ns, non-significant; UCPTB, uncomplicated pulmonary TB; CPTB, complicated pulmonary TB; LN-EPTB, lymph node extra-pulmonary TB; EN-EPTB, extra-nodal extra-pulmonary TB.

### IgG levels at diagnosis in pediatric TB disease

Elevated serum IgG for age and sex was displayed by 18/63 (28.6%) of UCPTB patients. The other three groups displayed significantly higher frequencies of elevated IgG when compared with UCPTB and similar rates among them: 60% in CPTB (15/25; *p* = 0.0079), 62.5% in LN-EPTB (5/8; *p* = non-significant), and 66.7% in EN-EPTB (10/15; *p* = 0.0142). No cases of hypogammaglobulinemia were identified in our cohort (Supplementary Table 1).

**Figure 1:**
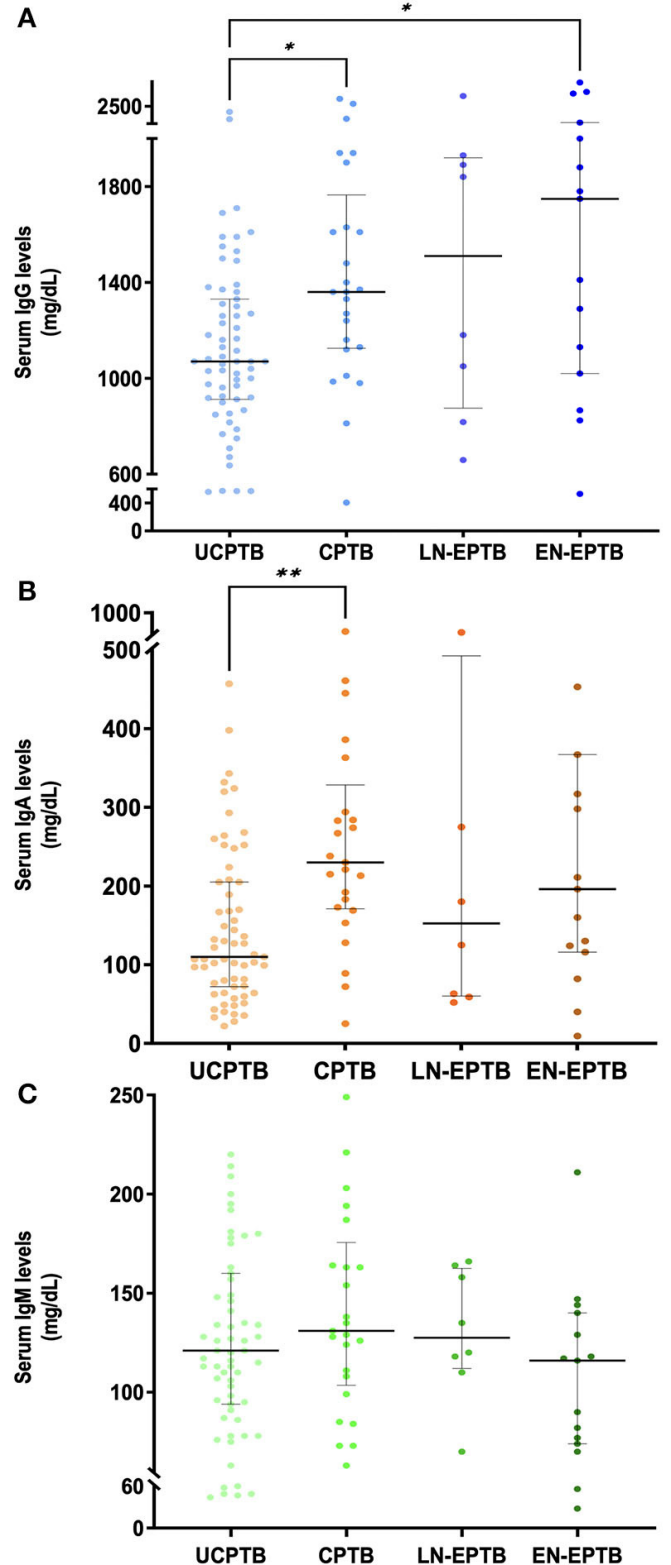
Serum Ig levels at diagnosis in patients with TB disease. **(A)** IgG, **(B)** IgA, and **(C)** IgM levels were screened at diagnosis in 111 patients, divided into the four groups, namely, UCPTB (*n* = 63), CPTB (*n* = 25), LN-EPTB (*n* = 8), and EN-EPTB (*n* = 15), according to TB severity and extent. Colored dots with different shades were employed to distinguish patients belonging to different TB groups. Kruskal-Wallis test was used to assess differences among the four TB study groups (*p < 0.05, **p < 0.01). Black horizontal bars indicate median levels, while vertical lines indicate interquartile range (IQR). Ig, immunoglobulins; TB, tuberculosis; UCPTB, uncomplicated pulmonary TB; CPTB, complicated pulmonary TB; LN-EPTB, lymph node extra-pulmonary TB; EN-EPTB, extra-nodal extra-pulmonary TB.

Overall comparison of median IgG levels revealed a significant difference among the 4 study groups (*p* = 0.0035). The lowest median serum IgG levels at diagnosis were detected in UCPTB (median, IQR: 1,070 mg/dl, 912–1,330). Progressively higher values were seen in CPTB (1,360 mg/dl, 1,125–1,765), LN-EPTB (1,510 mg/dl, 875–1,920), and EN-EPTB (1,748 mg/dl, 1,020–2,030) patients. In particular, IgG values were significantly lower in UCPTB, when compared with CPTB (*p* = 0.0245) and EN-EPTB (*p* = 0.0313) (Figure 1A).

### IgA levels at diagnosis in pediatric TB disease

Increased serum IgA for age and sex was observed only in 10/63 (15.8%) of UCPTB patients. The other three groups displayed higher frequencies of elevated IgA: 25% (2/8) in LN-EPTB, 36% (9/25) in CPTB, and 40% (6/15) in EN-EPTB (Supplementary Table 1). No patients displayed definitive SIgAD. Of note, three patients displayed probable SIgAD: two had UCPTB, while one presented with disseminated TB (EN-EPTB), although lacking other major immunologic defects.

As for IgG, the overall comparison of median serum IgA levels showed a meaningful difference among the four study groups (*p* = 0.0019). UCPTB patients displayed the lowest median serum IgA levels (median, IQR: 110 mg/dl, 72–205). CPTB subjects had the highest values of serum IgA (230 mg/dl, 171–328), followed by EN-EPTB (196 mg/dl, 116–367) and LN-EPTB (152 mg/dl, 60–482). In particular, IgA levels were significantly higher in complicated CPTB than in UCPTB (*p* = 0.0015) (Figure 1B).

### IgM levels at diagnosis in pediatric TB disease

Serum IgM at diagnosis exceeding ±2 SD was only found in one patient with EN-LNTB presenting with SIgMD confirmed in multiple determinations. Given the paucity of major alterations (>2 SD or < −2 SD) in serum IgM levels, we also analyzed frequencies of borderline high or borderline low (i.e., 1 < SD <2 and −1 < SD < −2, respectively) IgM values among the four study groups, as shown in Supplementary Table 1. In particular, the CPTB group had the highest proportion of patients with borderline-high IgM (4/25, 16%), while subjects with EN-EPTB most frequently displayed borderline-low IgM levels (7/15, 46%).

Median serum IgM levels at diagnosis in the different study groups were substantially similar, and no significant difference was detected (Figure 1C). The higher median values were detected in CPTB subjects (131 mg/dl, 103–175) followed by LN-EPTB subjects (127 mg/dl, 112–162), UCPTB subjects (121 mg/dl, 94–160), and EN-EPTB subjects (116 mg/dl, 74–140).

Of note, the patient with SIgMD presented at the age of 18 months with a chronic subcutaneous abscess of the right foot (Supplementary Figure 2) resistant to multiple antibiotic therapy and surgical drainage. MTB was identified on skin biopsy and further testing revealed a clinically silent pulmonary TB disease. The skin involvement was thus considered metastatic, and a diagnosis of tuberculous gumma was made. Extended immunological workup did not allow the identification of additional anomalies of the immune system, and SIgMD diagnosis was confirmed (Supplementary Table 3).

### IgG, IgA, and IgM levels during follow-up

**Figure 2:**
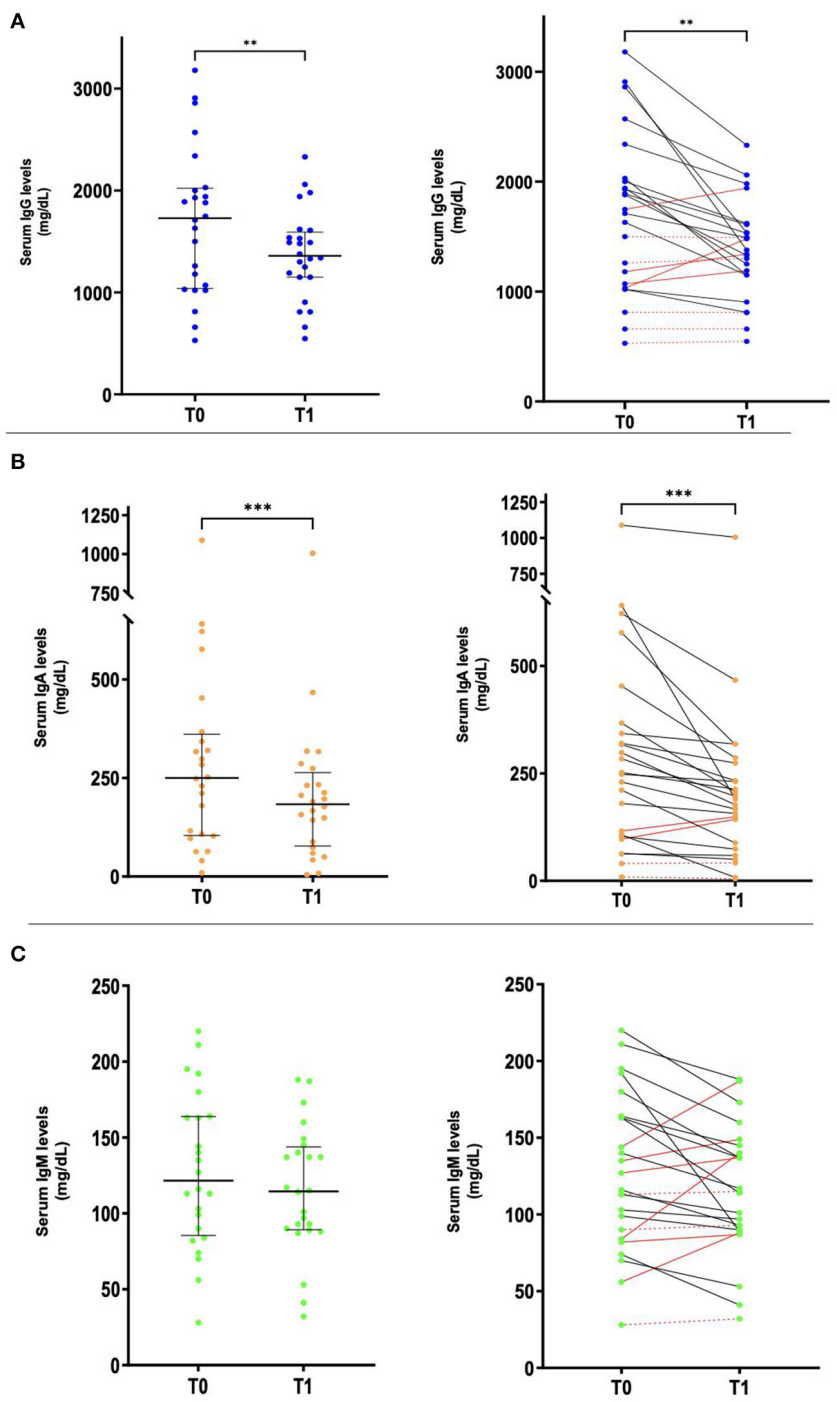
Variation in serum Ig levels from diagnosis (T0) to follow-up (T1). Left panels: scatter plots showing median **(A)** IgG, **(B)** IgA, and **(C)** IgM levels and IQR. Right panels: before-after comparison of serum **(A)** IgG; **(B)** IgA, and **(C)** IgM levels. Comparison between T0 and T1 was performed using *t*-test or Mann-Whitney test in case of normal or non-normal distributions, respectively (**p < 0.01, ***p < 0.001). In the left panels, black horizontal bars indicate median levels, while vertical lines indicate IQR. In the right panels, black lines indicate a reduction of serum IgG/IgA/IgM levels from T0 to T1, red continuous lines indicate an increase in serum Ig levels from T0 to T1, and red dotted lines indicate patients whose IgG/IgA/IgM levels were stable from T0 to T1. Ig, immunoglobulins; IQR, interquartile range.

**Figure 3:**
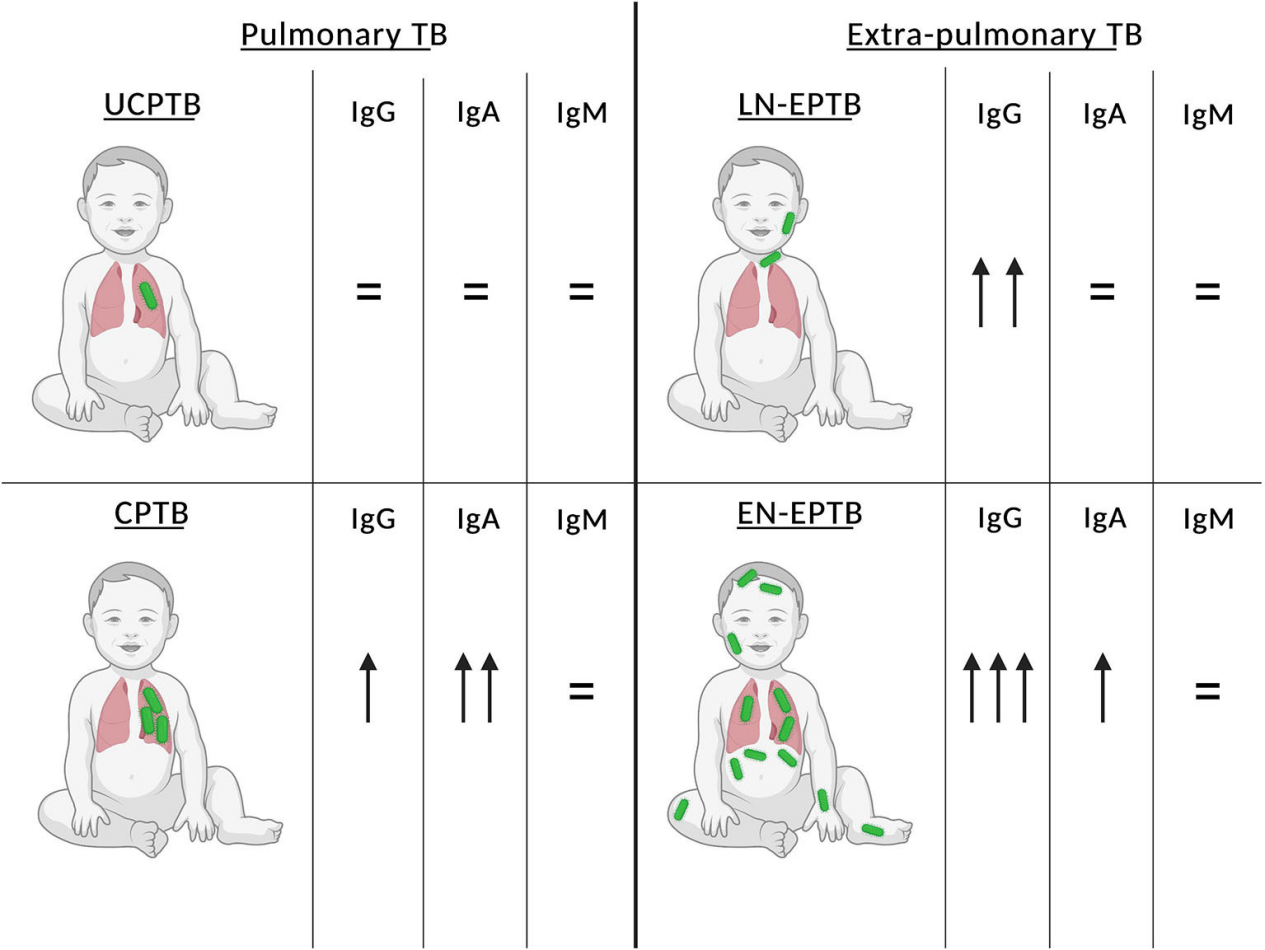
Patterns of serum IgG, IgA, and IgM levels at diagnosis in the four TB study groups. Median serum Ig levels in each TB study group were compared with median levels of sex- and age-matched reference values. Equal sign or upward arrows were assigned according to the difference between median values as follows: difference 0–0.5 SD (equal sign), 0.5–1 SD (one upward arrow), 1–2 SD (two upward arrows), and >2 SD (three upward arrows). TB, tuberculosis; SD, standard deviation; UCPTB, uncomplicated pulmonary TB; CPTB, complicated pulmonary TB; LN-EPTB, lymph node extra-pulmonary TB; EN-EPTB, extra-nodal extra-pulmonary TB.

A total of 24 children (median age: 11.4 years, IQR 3.6–16.1; 41.7% females) repeated serum Ig levels after 2.2 months (median; IQR 1.1–4.5) from treatment initiation. Serum Ig levels decreased from diagnosis to follow-up in 15/24 (63%) patients for IgG, 20/24 (83%) patients for IgA, and 15/24 (63%) patients for IgM (Figure 2). Median serum Ig values at diagnosis and follow-up are reported in Supplementary Table 2, showing a significant reduction in IgG (*p* = 0.0028) and IgA (*p* = 0.001) levels between these two time points. A specific analysis of serum Ig level modification during follow-up in each TB group could not be performed due to the limited sample size. Of note, IgA and IgM levels in patients with probable SIgAD (1 case analyzed at follow-up) and SIgMD (one case) respectively, did not change between diagnosis and follow-up. Finally, Figure 3 summarizes the findings of our study in a figurative model.

## Discussion

Children diagnosed with TB disease display significantly higher frequencies of elevated serum IgG and IgA and higher serum concentrations in complicated pulmonary and extrapulmonary forms when compared with uncomplicated cases with lung-restricted involvement. Serum IgM levels do not show significant variations across patient groups sorted by disease severity and extent of organ involvement. Moreover, elevated Ig values tend to regress during anti-TB chemotherapy, suggesting that they could represent an additional biomarker to monitor the response to treatment. Remarkably, reduced Ig levels at diagnosis revealed underlying primary disorders of humoral immunity (SIgMD and SIgAD), underlining that low Ig levels in TB should not be interpreted as immune alterations secondary to the ongoing infection.

Data on total serum Ig levels in TB disease are available only in adult subjects (17–25). These studies showed that serum IgG levels in adults with TB are unequivocally increased, while data about IgA and IgM seem contradictory. Several studies agree that IgA levels tend to be elevated, while IgM levels are usually in the normal range (17–20, 22, 23). On the contrary, increased IgM and/or normal IgA values during TB have also been reported (20, 21, 24). Correlations with disease extent are scarce, but more elevated levels of IgA (19) or both IgA and IgG (22) have been described in advanced pulmonary TB. Moreover, the scant available data regarding patients' follow-up show a general decrease in serum Ig levels (20), which may remain elevated in untreated patients (25).

Interestingly, serum Ig levels in our pediatric TB cohort exhibit a similar behavior. We divided our pediatric population into four study groups according to disease severity and organ involvement. Serum IgG levels at diagnosis progressively increase across the four groups: the lowest levels are displayed in uncomplicated cases (UCPTB) and the highest ones are seen in children with disseminated TB (EN-EPTB). Serum IgA levels show a similar pattern. Remarkably, our study confirmed a significant difference in terms of IgG and IgA levels between UCPTB and CPTB, as already seen in previous investigations on adult subjects (19, 22). Therefore, a higher serum IgG level at diagnosis may suggest a more severe TB disease. On the contrary, serum IgM levels did not differ among our four study groups. Of note, EN-EPTB cases display the highest frequency (7/15, 46%) of borderline-low IgM, revealing that IgM levels tend to behave oppositely to other Ig classes, being normal-low in disseminated TB cases. Finally, we confirmed the results of previous studies on adults, since also in children serum Ig levels are reduced upon anti-TB therapy (20, 25). Ig levels may therefore help in monitoring response to treatment, which must anyhow be decided in accordance with TB international guidelines (29).

Our clinical findings lack a definitive explanation at a cellular level, although several considerations can be made based on current knowledge of TB's immunology (3). Notably, MTB employs granuloma as a shelter to escape immune response (31). In such histological context, B lymphocytes locate at a peripheral site—away from infected macrophages (4)—where they may form B-cell follicles (BCFs) (7). Complicated forms of TB are instead characterized by a disruption of granuloma's architecture (32). The rupture of such immunological niche, together with elevated mycobacterial loads in tubercular cavities (32), may therefore boost both cellular and humoral immune responses, since MTB presents a meaningful immunostimulatory capacity (33). Dissemination of MTB outside of the lung may further enhance this process (34). Notably, tubercular cavitations are a sign of post-primary disease (32), and therefore, MTB proliferation induces a secondary humoral immune response that may explain the elevation of serum IgG despite normal levels of IgM (22, 35). Current evidence highlights that such IgG overproduction may target a wide variety of MTB antigens, with person-to-person heterogeneity (36). Moreover, contact of MTB with mucosal surfaces may lead to increased IgA production (37, 38). However, in our cohort of patients, the frequencies of elevated IgA and median IgA levels were not necessarily lower in nodal forms when compared with pulmonary ones that are supposed to have a greater proportion of mucosal involvement. Instead, the frequencies of elevated IgA were found to be progressively higher in LN-EPTB, followed by CPTB and reaching the higher rate in EN-EPTB. This may suggest that their elevation could be eventually related to the higher extent of activation of the immune system and of the inflammatory response encountered in more complicated and disseminated forms.

Given these premises, we may speculate that elevated IgG and IgA levels in patients with complicated and/or disseminated TB may correlate with the entity of the ongoing immune-mediated tissue damage triggered by MTB (39), as already seen for CD4+ Tcell depletion in children with disseminated TB disease (11, 40). Considering these findings, we hypothesize that severe forms of TB seriously impact on humoral and cellular immune responses. While the former is shifted toward an activated status, the latter is blunted, most likely due to an increased T lymphocyte pooling at the site of infection (11, 41). In a wider perspective, the concurrence of hypergammaglobulinemia and low T CD4+ lymphocytes in children reminds immunological findings in vertically acquired HIV infection (42, 43). Also in this context, children with severe forms and worse survival rates display more elevated Ig levels and low T CD4+ counts (44). Even though the infectious trigger is completely different, we cannot fail to point out that children with severe TB disease and HIV infection share similar humoral and cellular alterations that may conceal common immunopathogenic mechanisms (45–47).

Among our pediatric cohort, 4 patients were discovered to bear a previously unrecognized primary disorder of humoral immunity (one SIgMD and three SIgAD cases). Even though numbers are too less to draw any conclusion, the distribution of SIgAD patients across the four TB study groups (2/3 UCPTB, 1/3 EN-EPTB) did not particularly differ from the rest of the cohort. On the contrary, the only patient with SIgMD displayed tuberculous gumma, an unusual feature of cutaneous TB, due to metastatic spread from the lungs (48, 49). As a matter of fact, while SIgAD is not associated with increased susceptibility to *Mycobacteria* (50, 51), other cases of disseminated mycobacterial infections in patients with SIgMD are reported (13, 14, 52). Further studies and more reports of similar cases are needed in order to ascertain such association and to investigate a potential underlying immunopathogenic mechanism. Anyhow, our study shows that children with TB disease do not display any major (i.e., < −2 SD or >2 SD) alteration of IgM levels. Therefore, the detection of IgM deficiency in patients with TB disease should be a warning sign of an underlying IEI and must not be interpreted as an immune alteration secondary to the concurrent tubercular infection.

Our study has several shortcomings, mainly due to its retrospective nature. We measured Ig levels at the time of diagnosis, when patients were not necessarily at the same time point of the natural history of MTB infection. Moreover, less than one-third of the patients were screened for Ig levels at follow-up. These 24 subjects were identified retrospectively from the analysis of clinical records; therefore, we cannot exclude a selection bias of patients who underwent a second dosage of Ig levels. In addition, the scant sample size of certain study groups (e.g., LN-EPTB) reduced the statistical power of our analyses. Larger, prospective investigations of children with TB disease are needed in order to overcome these limitations and to assess any correlation between a specific Ig class and clinical or microbiological findings. Moreover, further studies involving immunological analysis on tubercular tissue specimens and/or extended immunophenotyping of peripheral lymphocytes may better clarify the role of B lymphocytes and humoral immunity in host response to MTB.

## Conclusion

We reported that TB in children significantly impacts on serum Ig levels, since both IgG and IgA values increase proportionally with the severity of lung disease and the extent of organ involvement. Healing from TB is associated with a reversion to normal IgG and IgA levels, which may therefore be useful clinical tools to evaluate response to treatment. Severely reduced Ig levels in children with TB disease may be a warning sign of an underlying IEI and should prompt clinicians to carry out an extended immunological workup. At a cellular level, several actors participate in host response to MTB, and the role of T lymphocytes and macrophages does not fully explain the complex immunologic scenario of TB. In this convoluted context, humoral immunity is not just a bystander.

## Data availability statement

The raw data supporting the conclusions of this article will be made available by the authors, without undue reservation.

## Ethics statement

Ethical review and approval was not required for the study on human participants in accordance with the Local Legislation and Institutional requirements. Written informed consent from the participants' legal guardian/next of kin was not required to participate in this study in accordance with the National Legislation and the Institutional requirements.

## Author contributions

FC, NC, SR, and LL conceptualized this study. FC and NC performed the data collection. EV, LB, and CM were responsible for patient recruitment and supplied patient care. SR, CC, FL, and LL analyzed the data. FC, NC, and LL wrote the original draft of the manuscript. LL, MG, EC, LG, and CA supervised the study. All authors contributed to the article and approved the submitted version.

## Conflict of interest

The authors declare that the research was conducted in the absence of any commercial or financial relationships that could be construed as a potential conflict of interest.

## Publisher's note

All claims expressed in this article are solely those of the authors and do not necessarily represent those of their affiliated organizations, or those of the publisher, the editors and the reviewers. Any product that may be evaluated in this article, or claim that may be made by its manufacturer, is not guaranteed or endorsed by the publisher.
