# Supplementary material for: Unbalanced serum immunoglobulins in clinical subtypes of pediatric tuberculosis disease
Source: Front Pediatr. 2022 Aug 9;10:908963. doi: 10.3389/fped.2022.908963 (PMC9395963; doi:10.3389/fped.2022.908963)
Supplement: Supplementary file 1 [file Data_Sheet_1.docx]

***Supplementary Material***

## Supplementary Figures

**SUPPLEMENTARY FIGURE 1 –** **Flow chart showing patients’ selection, included patients at diagnosis/at follow-up and their subdivision in the 4 TB study groups**.

**
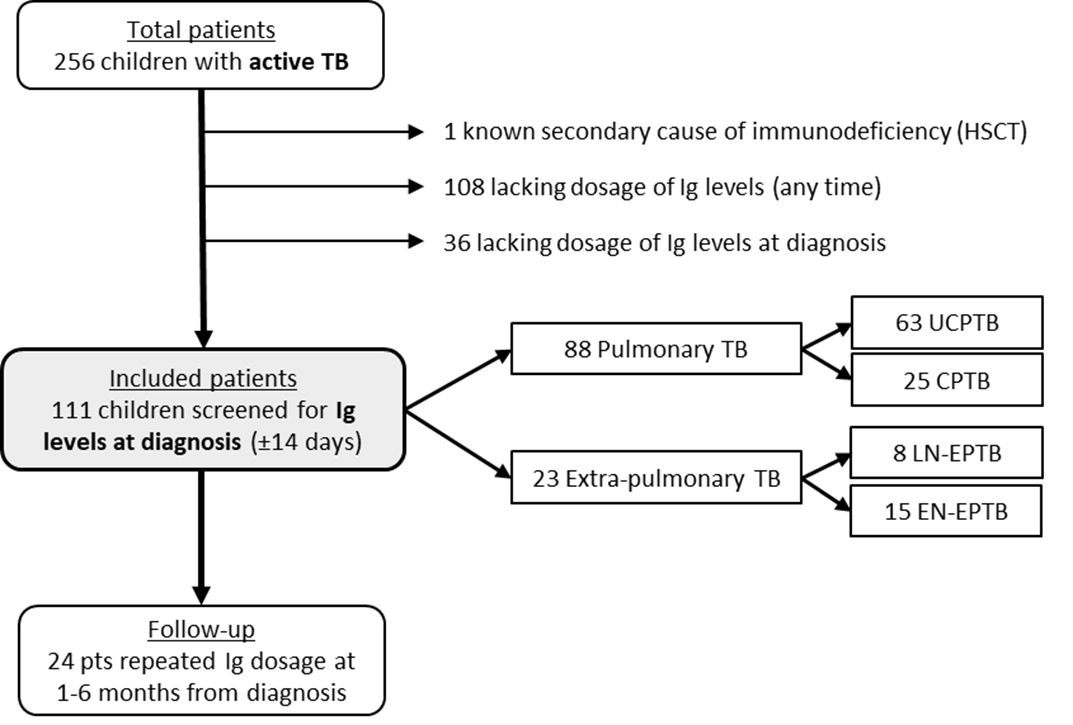
**

Abbreviations: CPTB, complicated pulmonary TB; EN-EPTB, extra-nodal extra-pulmonary TB; HSCT, hematopoietic stem cells transplantation; Ig, Immunoglobulins; LN-EPTB, lymph node extra-pulmonary TB; pts, patients; TB, tuberculosis; UCPTB, uncomplicated pulmonary TB.

**SUPPLEMENTARY FIGURE 2** – **Tuberculous gumma in a patient affected by selective IgM deficiency (SIgMD).** **A.** Clinical presentation as a swollen, painful red nodule on the upper surface of the right foot. **B**. Good clinical response after completion of a 6 month-long 4 drug anti-tuberculosis treatment regimen.


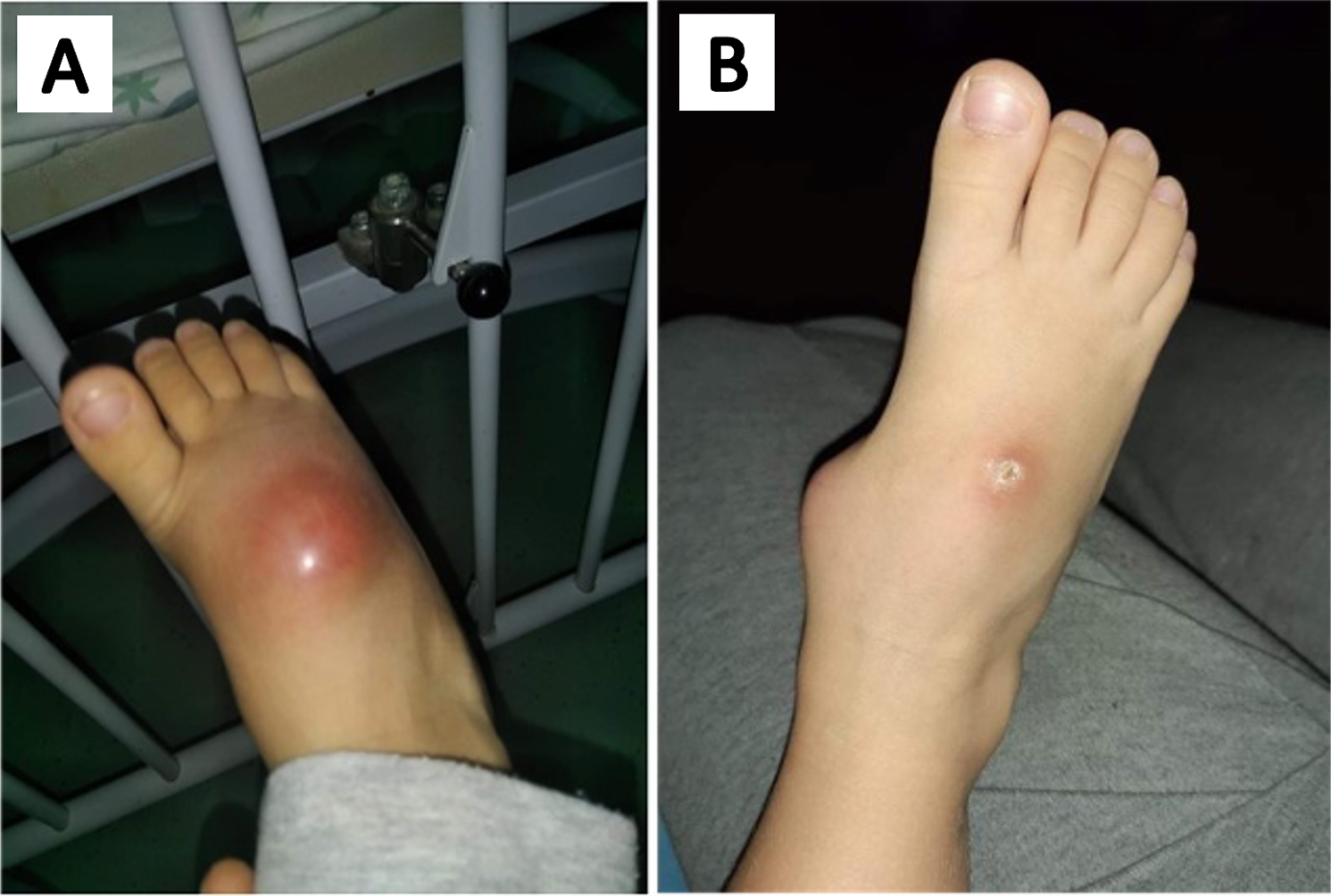


## Supplementary Tables

**SUPPLEMENTARY TABLE 1** - **Distribution of serum Ig levels for age and sex at diagnosis in each TB study group.**

| **Distribution of Ig levels %** | | **UCPTB** | **CPTB** | **LN-EPTB** | **EN-EPTB** |
| --- | --- | --- | --- | --- | --- |
|  |  | (n=63) | (n=25) | (n=8) | (n=15) |
| **IgG** | **Elevated** | 28.6 % | 60 % | 62.5 % | 66.7 % |
|  | **Normal** | 71.4 % | 40 % | 37.5 % | 33.3 % |
|  | **Reduced** | / | / | / | / |
| **IgA** | **Elevated** | 15.9 % | 36 % | 25 % | 40 % |
|  | **Normal** | 80.9 % | 64 % | 75 % | 53.3 % |
|  | **Reduced** | 3.2 % | / | / | 6.7 % |
| **IgM** | **Borderline high** | 14.3 % | 16 % | 12.5 % | / |
|  | **Normal** | 66.7 % | 68 % | 75 % | 53.3 % |
|  | **Borderline**  **low** | 19 % | 16 % | 12.5 % | 46.7 % |

Abbreviations: TB, tuberculosis; Ig, Immunoglobulins; n, number of patients; UCPTB, uncomplicated pulmonary TB; CPTB, complicated pulmonary TB; LN-EPTB, lymph node extra-pulmonary TB; EN-EPTB, extra-nodal extra-pulmonary TB.

**SUPPLEMENTARY TABLE 2** – **Demographic data and serum Ig levels at diagnosis (T0) and follow-up (T1).** Only patients whose Ig levels were available both at diagnosis and at follow-up were considered for the analysis.

| **Ig at follow-up** | **T0** | **T1** | | **p value** |
| --- | --- | --- | --- | --- |
|  | (n=24) | (n=24) | |  |
| **Female n°** | 10  (41.7%) | 10  (41.7%) | | / |
| **Sex ratio M:F** | 14:10 | 14:10 | | / |
| **Age years**  median (IQR) | 11.5 (3.6-16.2) | 11.6 (3.8-16.6) | | / |
| **IgG mg/dL**  median (IQR) | 1729 (1040-2023) | 1359 (1150-1592) | | 0.0028 |
| **IgA mg/dL**  median (IQR) | 250 (104-361) | 184 (78-264) | | 0.001 |
| **IgM mg/dL**  median (IQR) | 122 (86-164) | 115 (89-144) | | ns |
| **Ig variation at follow-up** | | | | |
| **IgG (%)** | Increased | | 4 (16.7) | |
|  | Stable | | 5 (20.8) | |
|  | Reduced | | 15 (62.5) | |
| **IgA (%)** | Increased | | 2 (8.3) | |
|  | Stable | | 2 (8.3) | |
|  | Reduced | | 20 (83.3) | |
| **IgM (%)** | Increased | | 6 (25.0) | |
|  | Stable | | 3 (12.5) | |
|  | Reduced | | 15 (62.5) | |

Abbreviations: F, female; ns, non-significant; Ig, immunoglobulins; IQR, interquartile range; M, male; n, number of patients.

**SUPPLEMENTARY TABLE 3** – **Extended immunological workup of the patient affected by selective IgM deficiency (SIgMD).**

| Lymphocyte subset | Distinctive flow cytometry markers | Absolute count /mmc  (%) | Reference range for age and sex (%) |
| --- | --- | --- | --- |
| B cells | CD19+  (% of total lymphocytes) | 1246  (27%) | 640-1960  (15-28)  Garcia-Prat 2018 |
| Transitional B cells | CD19+ CD24+  (% of B-cells) | 128  (10.3%) | 35-172  (5.2-17.2)  Ding et al, JACI, 2018 |
| Memory B cells | CD19+ CD27+  (% of B-cells) | 87  (7%) | 26-124  (3.0-14.2)  Ding et al, JACI, 2018 |
| Non-switched memory B cells | CD19+ CD27+ IgD+  (% of B-cells) | 58  (4.7%) | 0-100  (2-10)  Garcia-Prat 2018 |
| Switched memory B cells | CD19+ CD27+ IgD-  (% of B-cells) | 29  (2.3%) | 0-100  (1-11)  Garcia-Prat 2018 |
| T cells | CD3+  (% of total lymphocytes) | 3013  (65%) | 1850-5960  (52-77)  Garcia-Prat 2018 |
| T helper cells | CD3+ CD4+  (% of total lymphocytes) | 1803  (39%) | 1140-3800  (30-58)  Garcia-Prat 2018 |
| T cytotoxic cells | CD3+ CD8+  (% of total lymphocytes) | 1008  (22%) | 540-1970  (12-27)  Garcia-Prat 2018 |
| Recent thymic emigrants | CD3+ CD31+  (% of T-helper) | 1298  72% | 190-2600  (37-100)  Schatorje et al, Clinical Immunology, 2012 |
| Virgin T cells | CD45+RA  (% of T-helper) | 1424  79% | 472-1760  (46.1-84.4)  Ding et al, JACI, 2018 |
| Memory T cells | CD45+RO  (% of T-helper) | 379  21% | 227-844  (14.8-55.9)  Ding et al, JACI, 2018 |
| NK cells | CD3- CD16+ CD56+  (% of total lymphocytes) | 339  (7%) | 150-1330  (3-24)  Garcia-Prat 2018 |
| Additional immunological workup | | | |
|  |  |  | **Reference range for age and sex** |
| Lymphocytes proliferation test (PHA) | 91% | | > 70% |
| Lymphocytes proliferation test (anti CD3/anti CD28 beads + IL2) | 94% | | > 70% |
| DHR Test | **Spontaneous**  0 %  **Stimulated**  99 % | | < 5 %  > 90 % |
| IgG C. tetani  (IU/mL) | 0.52 | | ≥ 0.1 IU/mL |
| IgG C. diphtheria  (IU/mL) | 0.38 | | ≥ 0.1 IU/mL |

Abbreviations: DHR, dihydrorhodamine; Ig, immunoglobulins; IL, interleukin; IU, International Units; mL, milliliters; PHA, phytohemagglutinin.
